# Identification of two novel heterozygous variants of *SMC3* with Cornelia de Lange syndrome

**DOI:** 10.1002/mgg3.2447

**Published:** 2024-05-11

**Authors:** Zhi Lei, Xiaorui Song, Xuan Zheng, Yanhong Wang, Yingyuan Wang, Zhirong Wu, Tian Fan, Shijie Dong, Honghui Cao, Yuefang Zhao, Zhiyi Xia, Liujiong Gao, Qing Shang, Shiyue Mei

**Affiliations:** ^1^ Henan Key Laboratory of Children's Genetics and Metabolic Diseases Children's Hospital Affiliated to Zhengzhou University, Henan Children's Hospital Zhengzhou Children's Hospital Zhengzhou Henan China; ^2^ Department of Neonatal Medicine Henan Children's Hospital Zhengzhou Children's Hospital Zhengzhou Henan China; ^3^ Rehabilitation Center Henan Children's Hospital Zhengzhou Children's Hospital Zhengzhou Henan China; ^4^ Department of Medical Imaging Henan Children's Hospital Zhengzhou Children's Hospital Zhengzhou Henan China; ^5^ Department of Ophthalmology Henan Children's Hospital Zhengzhou Children's Hospital Zhengzhou Henan China; ^6^ School of Life Sciences Inner Mongolia University Hohhot Inner Mongolia China; ^7^ Department of Pediatric Intensive Care Unit Henan Children's Hospital Zhengzhou Children's Hospital Zhengzhou Henan China

**Keywords:** CdLS3, Cornelia de Lange syndrome, mosaic, *SMC3*

## Abstract

**Background:**

Cornelia de Lange syndrome (CdLS) is a multisystem genetic disorder, and cases caused by variants in the structural maintenance of chromosomes protein 3 (*SMC3*) gene are uncommon. Here, we report two cases of CdLS associated with novel pathogenic variants in *SMC3* from two Chinese families.

**Methods:**

Clinical presentations of two patients with CdLS were evaluated, and specimens from the patients and other family members were collected for Trio‐based whole‐exome sequencing. Pyrosequencing, chip‐based digital PCR, minigene splicing assay, and in silico analysis were carried out to elucidate the impact of novel variants.

**Results:**

Novel heterozygous variants in *SMC3* were identified in each proband. One harbored a novel splicing and mosaic variant (c.2535+1G>A) in *SMC3*. The mutated allele G>A conversion was approximately 23.1% by digital PCR, which indicated that 46.2% of peripheral blood cells had this variant. Additionally, in vitro minigene splicing analysis validated that the c.2535+1G>A variant led to an exon skipping in messenger RNA splicing. The other carried a heterozygous variant (c.435C>A), which was predicted to be pathogenic as well as significantly altered in local electrical potential. The former showed multiple abnormalities and marked clinical severity, and the latter mainly exhibited a speech developmental disorder and slightly facial anomalies.

**Conclusion:**

Both patients were clinically diagnosed with Cornelia de Lange syndrome 3 (CdLS3). The newly identified *SMC3* gene variants can expand the understanding of CdLS3 and provide reliable evidence for genetic counseling to the affected family.

## Graphical abstract

Identification of two novel potential pathogenic variants in *SMC3* in two clinically heterogeneous children with CdLS.

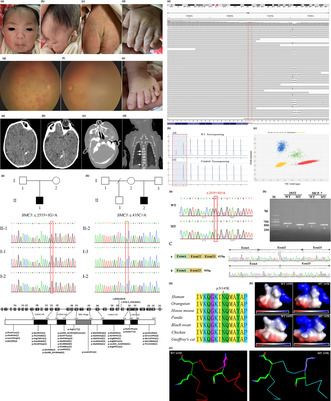

## 1 INTRODUCTION

Cornelia de Lange syndrome (CdLS) is a congenital developmental disorder typically involving physical, cognitive, and behavioral characteristics, which are inherited in an autosomal dominant or X‐linked manner. It is reported that the incidence of CdLS in newborns is 1/10000–1/30000 (Bottai et al., 2019). Pathogenic variants in genes encoding components of the cohesin complex (*NIPBL*, *SMC1A*, *SMC3*, *RAD21*, and *HDAC8*) are the primary causes of CdLS. Causative variants in other genes, such as *BRD4*, *KMT2A*, *EP300*, and *ANKRD11*, can led to a CdLS‐like phenotype (Kaur et al., 2023). About 70% of the affected individuals are related to *NIPBL* variants, and CdLS caused by structural maintenance of chromosomes protein 3 (*SMC3*) accounted for less than 1% of all cases (Kline et al., 2018).

The *SMC3* gene encodes a subunit of the evolutionarily conserved multimeric cohesin complex, which has been implicated in a wide range of functions, including sister chromatid cohesion, DNA repair mechanisms, gene expression regulation, and maintenance of genome stability (Gil‐Rodríguez et al., 2015). Previously, a proband with mild facial features and cognitive delay was identified with a de novo 3‐nucleotide (nt) deletion (c.1464_1466del) in *SMC3* from a screening of 115 *NIPBL*‐mutation‐negative individuals with CdLS or CdLS variant phenotypes. This was the first report of *SMC3* gene pathogenic variant causing Cornelia de Lange syndrome 3 (CdLS3, MIM #610759) (Deardorff et al., 2007). Subsequently, through clinical comparison of 16 patients with CdLS‐like features caused by variants in *SMC3* with typical CdLS, de novo *SMC3* variants were reported to account for 1%–2% of CdLS‐like phenotypes, and many SMC3‐associated phenotypes are also characterized by postnatal microcephaly but with a less distinctive craniofacial appearance, a milder prenatal growth retardation that worsens in childhood, few congenital heart defects, and an absence of limb deficiencies; though most variants are unique, two unrelated affected individuals shared the same variant but presented with different phenotypes (Gil‐Rodríguez et al., 2015). To date, there have been few clinical reports on individuals with *SMC3* variants, and the available clinical phenotypic information may be inadequate. Here, we report two cases of CdLS from two unrelated Chinese families and summarize the clinical and genetic characteristics of these CdLS3 patients.

## 2 MATERIALS AND METHODS

### 2.1 Ethical approval

All subjects provided signed informed consent forms for participation in the present study. The study was approved by the Ethics Committee of Zhengzhou Children's Hospital (Approval No. 2022‐K‐074).

### 2.2 Gene sequencing and analysis

Genomic DNA was extracted from peripheral blood samples using the Blood Genome DNA Midi Kit (CWBIO, China), according to the manufacturer's instructions. Trio‐based whole‐exome sequencing (Trio‐WES) was employed to detect the sequence variants in the probands and their parents. Exomes were captured with the xGen® Exome Research Panel v2 (IDT, USA) according to the manufacturer's protocol. DNA sequencing was performed on the NovaSeq 6000 platform (Illumina) with PE150. The reference genome was human GRCh37/hg19. The GATK v4.1.7 software was employed for variant calling, and variant annotation and interpretation were conducted using ANNOVAR software and the authorized Chigene Cloud platform (http://www.chigene.org). The pathogenicity of genetic variation was determined according to the American College of Medical Genetics and Genomics (ACMG) classification criteria and guidelines (Richards et al., 2015). Sanger sequencing was used to validate *SMC3* variants in the probands and their parents.

### 2.3 Pyrosequencing and digital PCR

Allelic quantification of mosaicism was carried out by pyrosequencing. Pyrosequencing analysis was conducted using a Vacuum Prep Workstation following the standard manufacturer's protocol (Qiagen, Germany). The allele quantification mode of PyroMark Q96 ID software was used for peak quantification. Digital PCR analysis was performed on QuantStudio™ 3D Digital PCR System (Thermo Fisher Scientific, USA) using specific primers and probes to detect the proportion of mosaic pathogenic variant. All primers and probes used are shown in Table 1.

**TABLE 1:** PCR primer sequence.

Target region	Primer sequence	Product length
*SMC3*_c.2535+1G>A	F‐ACATGAGCACAAGTACTAGAGAGG R‐ACACTAAGCTTTCTGTCCCCAAA	321 bp
*SMC3*_c.435C>A	F‐ATATTCCCATCATGGAGCCACTCT R‐GGAAATGCTTGTTGGAACATTTTGG	439 bp
*SMC3*_pyrosequencing	F‐ACAGACAGTTGCTAAATGAAAGAAT R‐Biotin‐GCCTCTGATATAACCACTTGCA S‐GCTTGGACCAAGTAGAACAG	181 bp
*SMC3*_cdPCR	F‐TCAATGAGAATCTGAGAAAACG R‐TGATATAACCACTTGCAATAGGT WT‐VIC‐CACATACACACCTGTTCTACTTG‐MGB Mut‐FAM‐CACATACACATCTGTTCTACTTG‐MGB	113 bp
*SMC3*_pcMINI‐C	F‐CTAGAGAACCCACTGCTTAC R‐TAGAAGGCACAGTCGAGG	613 bp (WT) 505 bp (MT)
*SMC3*_pcDNA3.1	F‐CTAGAGAACCCACTGCTTAC R‐TAGAAGGCACAGTCGAGG	586 bp (WT) 478 bp (MT)

### 2.4 Minigene splicing assay

Disruption of messenger RNA (mRNA) splicing prediction used splicing assessment algorithms such as RDDC, varSEAK, and SpliceAI. Further, in vitro minigene splicing assays were conducted to elucidate the impact of splicing. The intron and exons adjacent to the splicing site were amplified and cloned into minigene plasmid. PCR products from the proband with splicing mutant (MT) and the wild type (WT) were separately cloned into the pcDNA3.1 vector and the pcMINI‐C vector. Then, recombinant vectors were transfected into MCF‐7 and 293T cell lines, respectively. RNA was extracted from the cultured transfected cells and reversely transcribed into complementary DNA (cDNA). Subsequently, the splicing pattern was analyzed using PCR, agarose gel electrophoresis, and Sanger sequencing.

### 2.5 In silico analysis of missense variant

Various in silico tools were used to predict the effect of missense variant, including MutationTaster (http://www.mutationtaster.org/), sorting intolerant from tolerant (SIFT) (https://sift.bii.a‐star.edu.sg/), protein variation effect analyzer (PROVEAN) (https://www.jcvi.org/research/provean?species=human), polymorphism phenotyping v2 (PolyPhen‐2) (http://genetics.Bwh.harvard.edu/pph2/), and rare exome variant ensemble learner (REVEL) (https://sites.google.Com/site/revelgenomics/). Meanwhile, the crystal structure (PDB ID: 6WG3) was chosen as the template, and the three‐dimensional structure visualization tool PyMOL was utilized to evaluate the changes in the protein structure after the missense variant.

## 3 RESULTS

### 3.1 Case presentations

**FIGURE 1:**
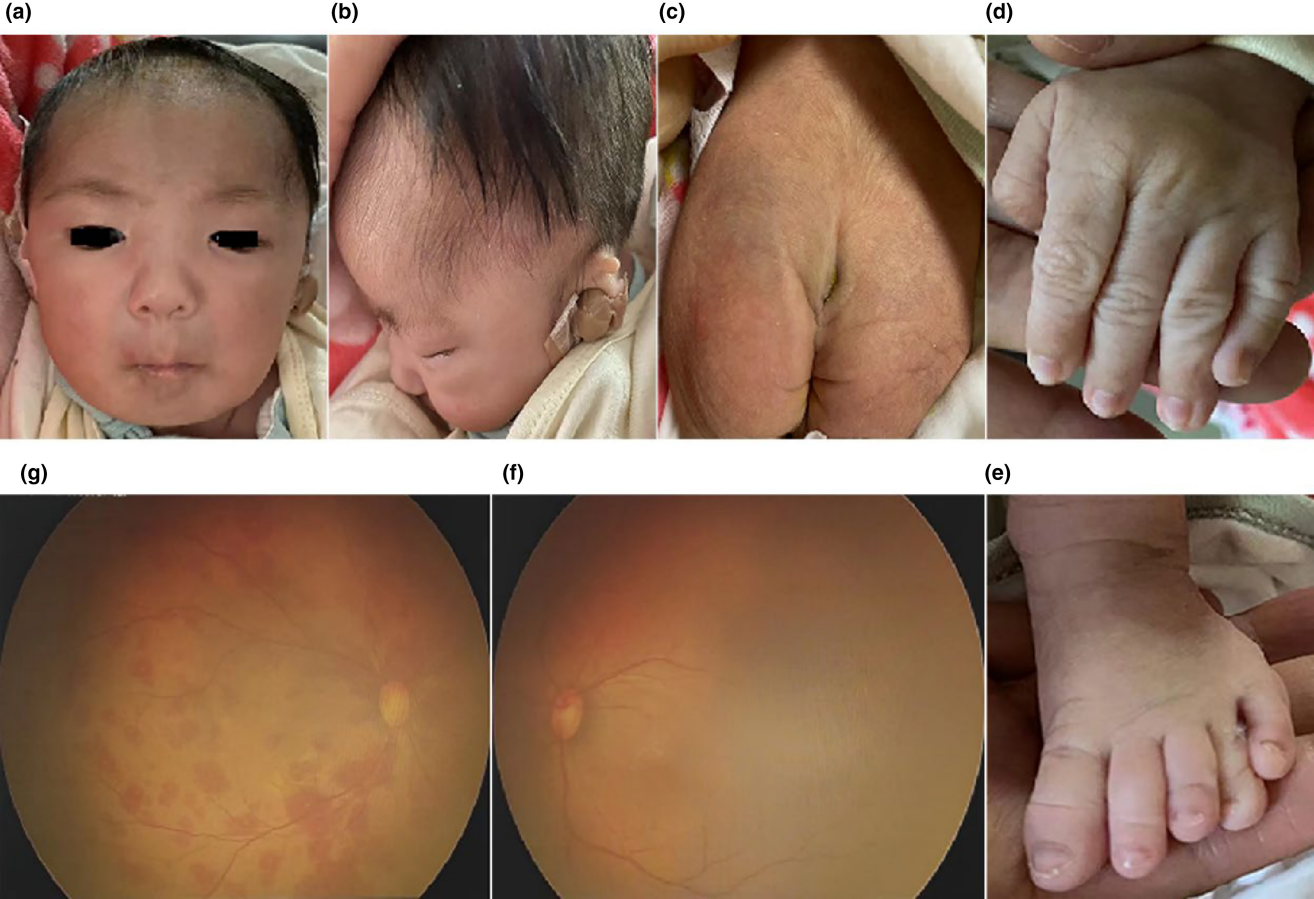
Main clinical features of Proband 1. (a) Facial phenotypes: thick eyebrows, low and flat nasal bridge, long philtrum, and micrognathia. (b) Left ear deformity. (c) Hirsute back and sacrococcygeal recess. (d) Clinodactyly of the 5th finger and (e) the 5th toe. (f) Left and (g) right eye fundus (extensive Roth spots).

**FIGURE 2:**
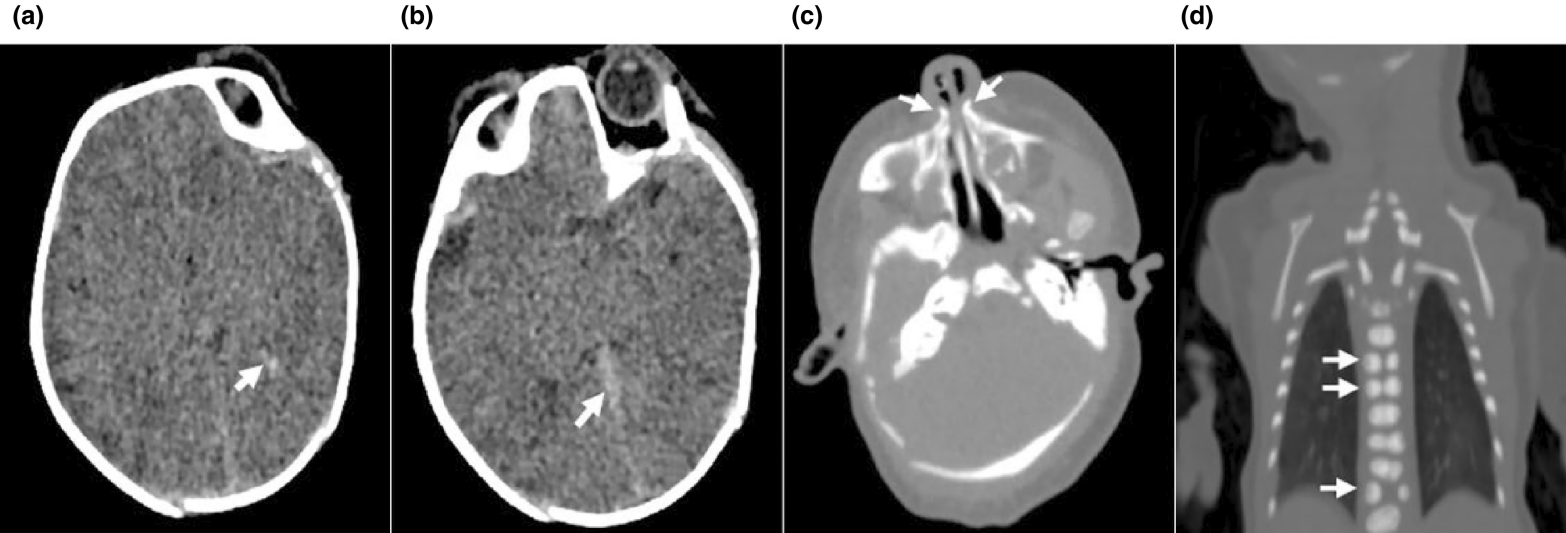
Computed tomography (CT) examination result of Proband 1. (a,b) Craniocerebral CT image of the patient's head, showing unclear demarcation between cerebral hemisphere gray and white matter and basal ganglia, linear high density seen in the posterior horn and posterior interhemispheric fissure of the left ventricle (arrow). (c) CT of the nasopharynx showing pyriform foramen stenosis (arrows). (d) CT of the thoracic spine showing increased bone density in the ribs, bilateral scapulae, and thoracic spine, and many vertebral bodies showed “sphenoid” deformity (arrows).

Proband 1 (P1) (Figure 4a II‐1), a 1‐day‐old male infant, was the first child of non‐consanguineous healthy parents without a family history of genetic diseases. He was born at term with a birth weight of 2.85 kg, a birth length of 47 cm, and a head circumference of 33 cm. After birth, he was admitted to the Neonatal Intensive Care Unit (NICU) because of respiratory distress, characterized by shortness of breath, wheezing, spitting, and dysmorphic features. Physical examination revealed thick eyebrows, a low broad nasal bridge, long philtrum, micrognathia, left ear deformity, short penis, foot varus, and slight clinodactyly of the fifth finger and toe (Figure 1a–e). Computed tomography (CT) of the head, nasopharynx and chest showed unclear boundaries of the gray and white matter and basal ganglia in the cerebral hemispheres, subarachnoid hemorrhage, a small amount of blood accumulation in the left ventricle, nasal piriform aperture stenosis and short mandible, part of the thoracic spine was deformed, and generalized increased bone density in the ribs, bilateral shoulder blades and thoracic spine (Figure 2). Thoracoabdominal radiograph suggested pneumonia, and the intestinal tract contained less gas. The cardiac ultrasound showed patent ductus arteriosus and patent foramen ovale. The abdominal ultrasound showed hepatomegaly, 35.3 mm subcostal. The hearing test of the left ear showed hearing impairment, while the right ear passed. Fundus examination revealed extensive Roth spots in the right eye but no obvious abnormalities in the left eye (Figure 1f,g). P1 was discharged at 3 days of age and died shortly after.

Proband 2 (P2) (Figure 4b II‐2) was a 4‐year‐4‐month‐old boy from a non‐consanguineous family. He was admitted to the rehabilitation department with the complaint of “poor speech.” Although he could speak simple words and sentences and engaged in simple questions and answers, his pronunciation was unclear. He was prone to hyperactivity, attention deficit, poor name‐calling responses, and irritable and aggressive behavior with autistic‐like features. The duration of eye contact with other people was short and showed timidity with strangers. The fine motor skills of both hands were not flexible and lacked coordination. Physical examination showed clear consciousness, normal mental response, low and flat nasal bridge, no edema of both eyelids, normal eye movement, and hypertelorism. Proband 2 was delivered by cesarean section due to premature rupture of amniotic fluid, with a birth weight of 2.55 kg. There were no abnormalities of the placenta or umbilical cord, no history of hypoxia or asphyxia, and jaundice persisted until 1 month of age. During rehabilitation treatment, the auditory brainstem response test showed abnormal auditory pathways in this child. According to the China Developmental Scale for Children (CDSC) (Chun‐Hua et al., 2014), the average cognitive age of P2 was 36 months, and the developmental quotient was 69 (normal range:85–114). The test for language retardation based on sign‐significant relations (S‐S method) (Yao et al., 2020) revealed that the child's language expression and comprehension were abnormal. In addition, Autism Behavior Checklist (ABC) and Social Responsiveness Scale (SRS) assessment suggested suspicious autistic‐like symptoms. Finally, he was clinically diagnosed with language delay and suspected of hereditary metabolic disease.

### 3.2 Genetic test results

**FIGURE 3:**
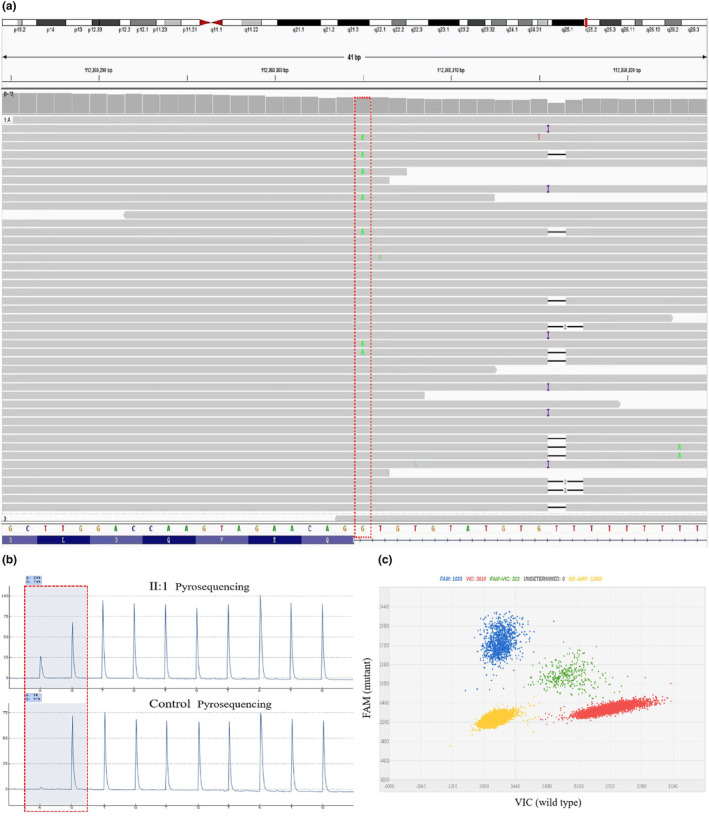
Mosaic pathogenic variants in *SMC3* of Proband 1 identified by Trio‐WES, pyrosequencing, and chip‐based QuantStudio 3D digital PCR. (a) A suspected mosaic splicing pathogenic variant was discovered by Trio‐WES, accounting for approximately 13.6% of the reads (red box). (b) Pyrosequencing confirmed the G>A substitution with a variation frequency of 26%. (c) Digital PCR analysis showed the presence of mutant (blue) and wild‐type (red) alleles, with a proportion of about 23.1%. Green cluster represents wells containing both VIC and FAM dyes, and the yellow cluster represented unamplified wells.

**FIGURE 4:**
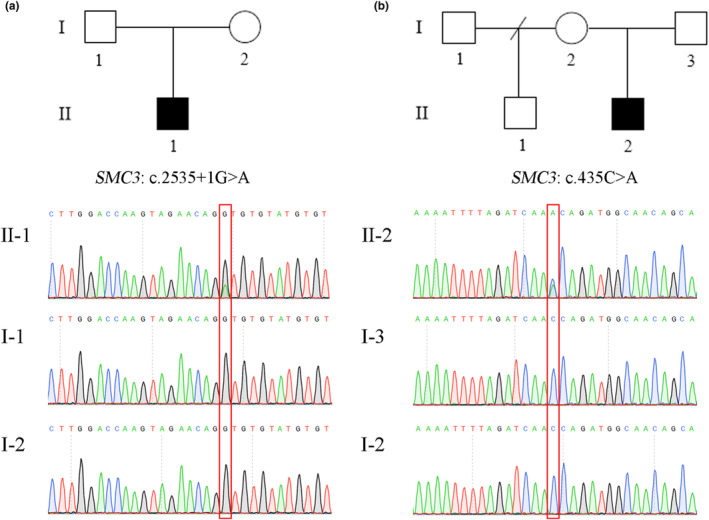
Pedigree of patients and the verification results of Sanger sequencing. (a) Pedigree of Proband 1(P1), and Sanger sequencing detected a heterozygous variant c.2535+1G>A of P1, which was not detected in either parent. (b) Pedigree of Proband 2(P2), and Sanger sequencing detected a heterozygous variant c.435C>A of P2, which was not detected in either parent.

A splicing *SMC3* variant (c.2535+1G>A, NM_005445.4) was identified in the site of intron 22 in P1 using Trio‐WES. The splice mutation was found in 13.6% (6/44) of the reads (Figure 3a). The G>A conversion was confirmed by pyrosequencing in which the mutated allele was approximately 26.0% compared with control (Figure 3b). Digital PCR analysis showed the presence of MT and WT alleles, with a proportion of about 23.1% (Figure 3c). Based on the results of Trio‐WES, P2 also carried a heterozygous missense variant in *SMC3* (c.435C>A/p.Asn145Lys). Sanger sequencing was used to verify that no variant was detected in either parent (Figure 4a,b). Additionally, the two variants were not found in the Exome Aggregation Consortium (ExAC October 2016, http://exac.broadinstitute.org), Genome Aggregation Database (gnomeAD v4.0, https://gnomad.broadinstitute.org/), 1000 Genome Project phase 3 (http://www.1000genomes.org/home), and Human Gene Mutation Database (HGMD Professional 2023.4, http://www.hgmd.cf.ac.uk/ac/index.php), suggesting that these were novel variants. According to the ACMG guidelines, variants c.2535+1G>A (PVS1_Strong+PS2_Moderate+PM2_Supporting) and c.435C>A (PS2_Moderate+PM1+PM2_Supporting+PP2+PP3) were classified as likely pathogenic.

### 3.3 Effect of *SMC3* : c.2535+1G>A variant on splicing

**FIGURE 5:**
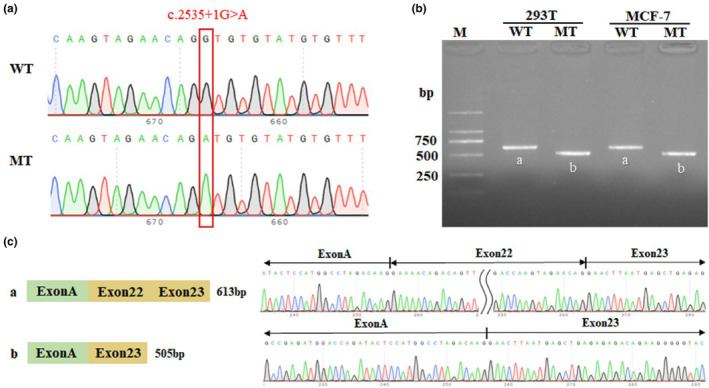
Minigene assay analysis of the *SMC3* variant c.2535+1G>A. (a) Sanger sequence of minigene in WT (above) and MT (below) group (c.2535+1G>A). (b) Agarose gel electrophoresis of RT‐PCR fragments showed the amplicon size in the MT group was smaller than the WT. (c) Sanger sequencing results of the WT and MT indicating the c.2535+1G>A variant caused the Exon22 skipping.

Splicing assessment algorithms (RDDC, varSEAK, and SpliceAI) suggested that the variant c.2535+1G>A affected the “mutation‐I region” of splicing, which might lead to abnormal splicing of exons. As P1 died in the neonatal period, fresh blood samples could not be obtained for RNA transcription level verification, so we performed an in vitro minigene validation experiment. Both WT and MT minigenes were respectively transfected into MCF‐7 and 293 T cells to verify this result (Figure 5a). The length of the product detected using reverse transcription PCR (RT‐PCR) in the WT group was consistent with the expected value (613 bp) in both MCF‐7 and 293 T cells. However, the amplicon size in the MT group was smaller than the WT (Figure 5b). Furthermore, Sanger sequencing of RT‐PCR products showed that the variant c.2535+1G>A affected the normal splicing of *SMC3* mRNA, resulting in an exon‐skip at Exon 22 (Figure 5c). Exon 22 skipping did not change the downstream reading frame but resulted in an internal deletion of 36 amino acids in SMC3. Its expression pattern at cDNA and protein level was c.2428_2535del (p.Asn811_Glu846del).

### 3.4 In silico analysis of c.435C>A

**FIGURE 6:**
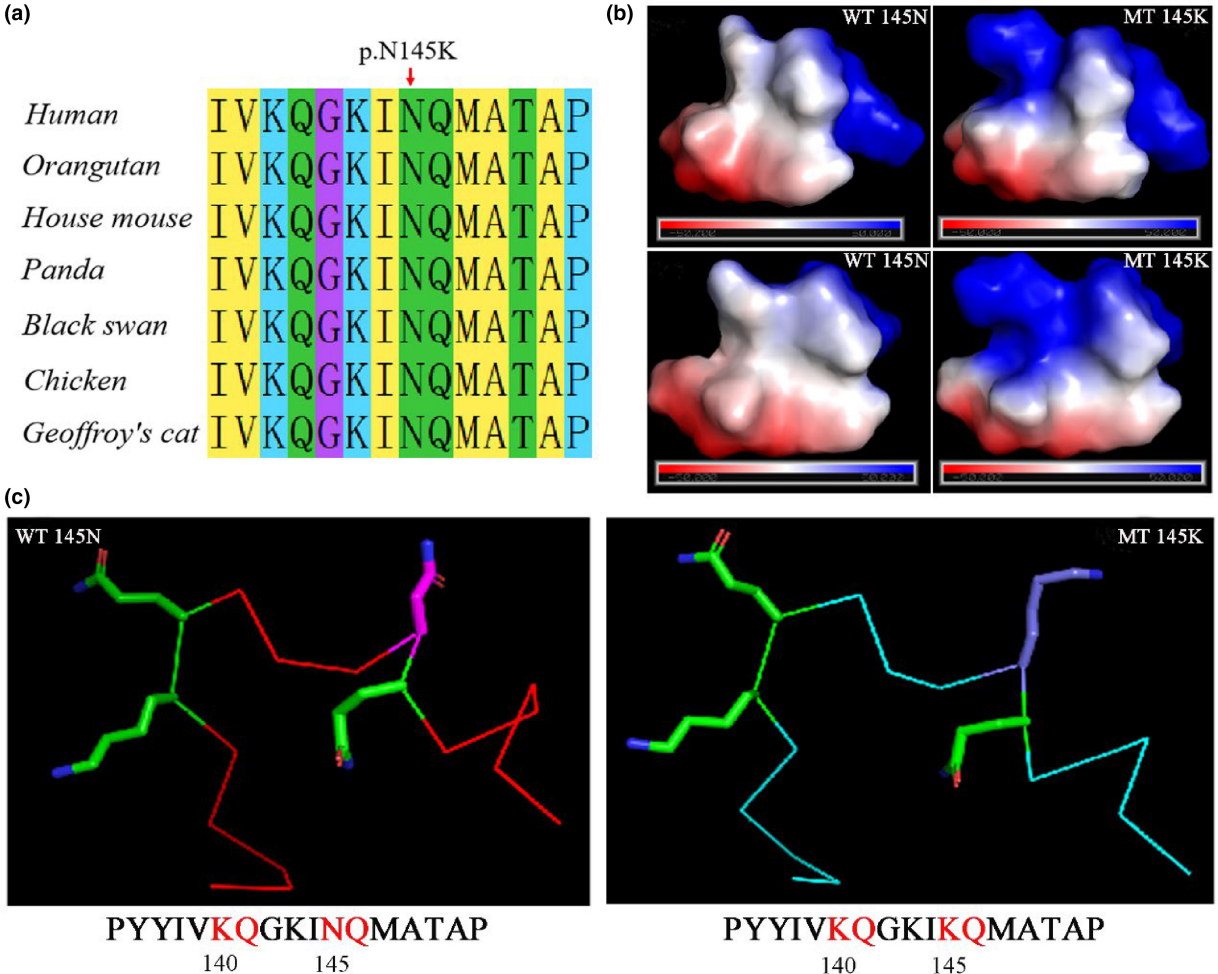
The impact of *SMC3* pathogenic variant c.435C>A on the protein's molecular structure. (a) Multiple alignments of SMC3 from different species revealed that amino acid residue 145 was highly conserved. (b) The local electrical potential map showed the change in local electrical potential before and after the mutation. (c) The prediction of acetylation modification changes in wild‐type and mutant sites of SMC3 protein.

The c.435C>A variant in exon 8 resulted in amino acid substitution at position 145 (N145K). Evolutionary conservation analysis revealed that amino acids at residue 145 were highly conserved in orangutans, mice, pandas, black swans, chickens, and cats (Figure 6a). All protein function prediction software tools predicted the c.435C>A mutation to be damaging. Compared with the WT protein, the N145K mutation was not perceived as a significant change in the overall complex. The local electrical potential map showed a significant change after the mutation (Figure 6b). In addition, we found that the N145K mutation (mutated sequence PYYIV**KQ**GKI **KQ**MATAP) was structurally similar to the sequence at position 140 (original sequence PYYIV**KQ**GKI **NQ**MATAP) (Figure 6c). It is hypothesized that this pathogenic variant is likely to affect the acetylation modification of lysine at position 140.

## 4 DISCUSSION

CdLS is a rare genetically heterozygous multisystem developmental disorder with highly variable clinical manifestations. Typical CdLS can be easily recognized from birth by experienced pediatricians and clinical geneticists, due to a distinctive craniofacial appearance as well as growth pattern and limb malformations (Kline et al., 2018). However, CdLS3 usually presents atypical clinical features and is not easily diagnosed clinically, which requires molecular genetic testing.

**FIGURE 7:**
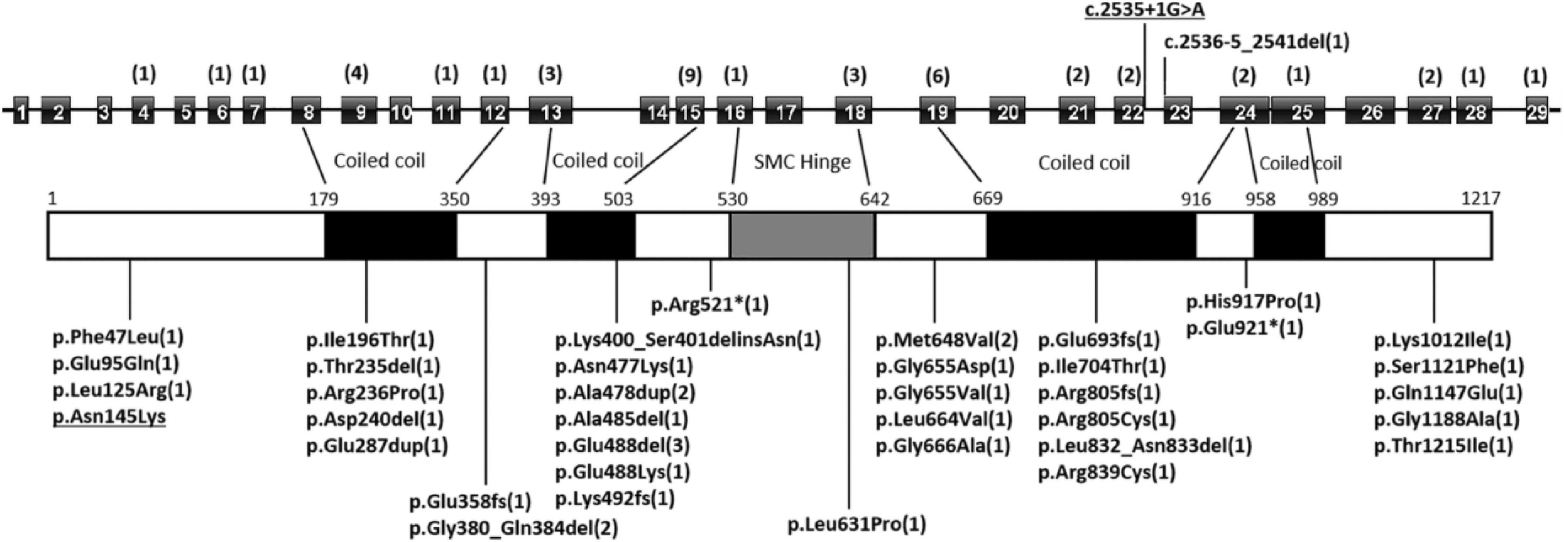
Summary of the individuals with *SMC3* pathogenic variants. The underlined part is the variation in this report. Numbers in brackets represent the number of cases in each exon region and variant, respectively.

An online database search was conducted on PubMed and Web of Science databases using “*SMC3*” and “Cornelia de Lange syndrome” as the keywords. The search yielded 45 cases of CdLS, with definite variants of *SMC3* reported as of July 2023, of which 24 cases had detailed clinical data (including the two cases in our study) (Figure 7) (Ansari et al., 2014; Bowling et al., 2017; Deardorff et al., 2007, 2012; “Deciphering Developmental Disorders Study”, 2017; Dowsett et al., 2019; Gao et al., 2019; Gil‐Rodríguez et al., 2015; Infante et al., 2017; Kaur et al., 2016; Kosmicki et al., 2017; Kruszka et al., 2019; Li et al., 2021; Liu et al., 2020; Rentas et al., 2020; Retterer et al., 2016; Sanders et al., 2012; Stanley et al., 2020; Stessman et al., 2017; Stranneheim et al., 2021; Turner et al., 2019; Yuan et al., 2015, 2019). No instances of these cases were observed within consanguineous families. The male‐to‐female ratio of CdLS3 was approximately 15:9. Approximately 25.0% (6/24) of the patients were adults and 41.7% (10/24) were children under the age of 3 years, including one fetus terminated at 21 weeks. The remaining 8 patients were aged between 3 and 18 years. Referring to the summary of clinical features of CdLS3 by Li et al. (2021), we found that all patients exhibited symptoms of verbal development delay and intellectual disability to varying degrees. Other relatively common clinical manifestations include arched eyebrows (86% [18/21]), long eyelashes (82% [18/22]), hirsutism (81% [17/21]), feeding problems in infancy (76% [13/17]), syndactyly of toes (75% [15/20]), and broad/bulbous nasal tip (74% [14/19]). The genetic characteristics of the condition were such that, as confirmed by the parents, except for one case in which the variant was inherited from the mother, 66.7% (16/24) of patients had de novo variants. Among 24 CdLS3 patients, half of the genetic variations were missense pathogenic variants, followed by deletions and duplications (10/24). Additionally, one case of nonsense pathogenic variant and one case of classic splicing pathogenic variant have been reported (the present report).

Our literature review showed the largest number of *SMC3* variants was in exon 15, accounting for 20% (9/45) of all reported variants. Other variants were found in exons 9, 13, 18, 19, 21, and 22 (20/45), which were in the coiled helix domain of SMC3 protein (Figure 7). In these cases, it was found that three patients had the same variant (c.1464_1466del); however, two of them with detailed information differ in clinical severity, where one case was assessed as mild and the other as moderate (Deardorff et al., 2007; Gil‐Rodríguez et al., 2015). The former had mild intellectual disability, while the latter showed severe intellectual disability. In addition, Infante et al. (2017) reported a mother and her daughter carrying the same *SMC3* variant (c.1433_1435dup), and the mother, who had mild symptoms, was not diagnosed until the pathogenic variant was identified in her child. The affected child had clinical features such as microcephaly, arched thick eyebrows, hirsutism, synophrys, mild ptosis, flat nasal bridge, a small chin with mild retrognathia, proximally placed thumbs, hypotonia, feeding difficulties, and growth retardation. However, when her mother underwent genetic testing at the age of 22, speech delay and learning disabilities were detected, with no evidence of growth retardation. The two patients exhibited varying degrees of clinical symptoms, indicating the phenotypic heterogeneity caused by *SMC3* variants.

In the current study, due to the special appearance (left ear deformity and foot varus) in P1, it was suspected that P1 might have a genetic disorder. Trio‐WES detection showed that there was a novel heterozygous splice site variant c.2535+1G>A in *SMC3*, which was not detected in either parent. According to the description and diagnostic criteria of CdLS (Deardorff et al., 2020), the clinical manifestations of P1 are considered to correlate with CdLS. The reanalysis of the original data of other CdLS‐associated genes *NIPBL*, *RAD21*, *BRD4*, *HDAC8*, and *SMC1A* revealed no suspected pathogenic variants. We also detected a heterozygous missense variant in P2, which was a de novo and unreported previously. Multiple algorithms (MutationTaster, PROVEAN, SIFT, PolyPhen‐2, and REVEL) predicted that the variant c.435C>A of P2 is deleterious.

P1 is the first report of a classic splicing variant in the *SMC3* gene, which had been confirmed as mosaicism of causative mutation. One of the previously reported patients was found to be mosaic in *SMC3* but her phenotype was classified as moderate according to the classification of the disease's severity proposed by Kline et al. (2007) and Ansari et al. (2014). Reports on CdLS mosaic patients concentrate mainly on the *NIPBL* gene, whose somatic mosaicism is not consistently connected with a milder phenotype (Ansari et al., 2014; Huisman et al., 2013). According to the reported cases, the onset, severity, and systemic involvement of mosaic mutation are influenced by the location of mutated cells and the extent of the pathogenic variant. Additionally, in vitro minigene splicing analysis showed that exon 22 skipping resulted in an internal deletion of 36 amino acids in SMC3. Two of the previously reported cases of CdLS3 were caused by mutations located in exon 22 of the *SMC3* (c.2494_2499del, c.2515C>T), with clinical severity ranging from moderate to severe (Gil‐Rodríguez et al., 2015). Ansari et al. demonstrate that individuals with heterozygous, predicted loss‐of‐function variants in *SMC3* are survivable, and phenotypes associated with variable developmental delay, growth deficiency, and/or facial dysmorphism, that are milder than but overlapping with that of *SMC3* missense/in‐frame indel variants present in CdLS cohorts (Ansari et al., 2024). Therefore, we strongly suspect that this variant (c.2535+1G>A) in vivo is the cause of most of the findings in P1. Moreover, additional studies revealed the presence of multiple abnormalities involving systems and organs, including patent ductus arteriosus, intracranial hemorrhage, congenital thoracic vertebral malformation, nasal piriform aperture stenosis, extensive Roth spots in the fundus of the right eye, left‐sided hearing loss, and hepatomegaly. The clinical presentation of these anomalies was markedly severe. Neither congenital nasal pyriform aperture stenosis nor thoracic vertebral malformation are commonly seen in CdLS and could be due to either this reported variant or another potential unknown genetic cause.

The c.435C>A variant in exon 8 causes a missense pathogenic variant at position 145, and it is believed that this pathogenic variant could lead to functional modifications. Compared with the WT protein, the neutral asparagine was changed to a positively charged lysine, which resulted in significant changes in the local electrical potential. Interactions with other subunits and negatively charged nucleic acids may be affected, which may impact the biological functions of the SMC3 protein. Additionally, previous studies have identified the presence of N6 acetylation modifications at lysine residues 105, 106, and 140 of SMC3. The modifications at these sites regulate the functioning of SMC3, and pathogenic variants of the corresponding sites to arginine were lethal in yeast studies (Choudhary et al., 2009; Shi et al., 2020).

## 5 CONCLUSION

In summary, we identified two novel variants in *SMC3*, which may constitute potential pathogenic variants associated with CdLS3. The clinical presentation of two cases was heterogeneous, in which one case showed multiple abnormalities and marked clinical severity, while the other mainly exhibited language delay and mild facial dysmorphic features. This further confirms previous research that loss‐of‐function variants in *SMC3* may lead to a more severe clinical phenotype. We also reviewed the literature on *SMC3* variants to further correlate genotype with CdLS phenotype, to provide an in‐depth comprehension of the function of this gene.

## AUTHOR CONTRIBUTIONS

Zhi Lei and Xiaorui Song designed and drafted the manuscript. Xuan zheng and Yanhong Wang organized the genetic analysis and bioinformatic analysis. Yingyuan Wang, Zhirong Wu, Tian Fan, Shijie Dong, and Honghui Cao collected the clinic data. Yuefang Zhao, Zhiyi Xia, and Liujiong Gao interpreted the laboratory test results. Shiyue Mei critically revised the manuscript. All authors reviewed and approved the manuscript.

## FUNDING INFORMATION

This work was supported by National Natural Science Foundation of China (81701125), Natural Science Foundation of Henan Province of China (232300421289), and Health Commission of Henan Provincial (Wjlx2022148) for funding the study.

## CONFLICT OF INTEREST STATEMENT

The authors declare no conflict of interest.

## ETHICS APPROVAL AND CONSENT TO PARTICIPATE

All subjects provided signed informed consent forms for participation in the present study. The present study was approved by the Ethics Committee of Zhengzhou Children's Hospital (Approval No. 2022‐K‐074).

## Data Availability

The data that support the findings of this study are available from the authors upon reasonable request and with permission of the Institutional Review Board of Zhengzhou Children's Hospital.
